# Dry Blood Spots a Reliable Method for Measurement of Hepatitis B Viral Load in Resource-Limited Settings

**DOI:** 10.1371/journal.pone.0166201

**Published:** 2016-11-07

**Authors:** Kathrine Stene-Johansen, Nadeem Yaqoob, Joakim Overbo, Hanna Aberra, Hailemichael Desalegn, Nega Berhe, Asgeir Johannessen

**Affiliations:** 1 Department of Virology, Norwegian Institute of Public Health, Oslo, Norway; 2 Medical Department, St. Paul’s Hospital Millennium Medical College, Addis Ababa, Ethiopia; 3 Aklilu Lemma Institute of Pathobiology, Addis Ababa University, Addis Ababa, Ethiopia; 4 Centre for Imported and Tropical Diseases, Oslo University Hospital, Ullevål, Oslo, Norway; 5 Department of Microbiology, Vestre Viken HF, Bærum, Norway; Centre de Recherche en Cancerologie de Lyon, FRANCE

## Abstract

**Background & Aims:**

Hepatitis B virus (HBV) quantification is essential in the management of chronic hepatitis B, both to determine treatment eligibility and in the monitoring of treatment effect. This test, however, is rarely available in resource-limited settings due to high costs and stringent requirements for shipment and storage of plasma. Dried Blood Spots (DBS) can be a convenient alternative to plasma, but its use for HBV monitoring has not been investigated under real-life conditions in Africa.

**Methods:**

The performance of DBS in HBV quantification was investigated using a modified commercial test (Abbott RealTime HBV assay). Paired DBS and plasma samples were collected from an HBV positive cohort in Addis Ababa, Ethiopia. DBS were stored at ambient temperature for 4–39 days before shipment to the laboratory.

**Results:**

Twenty-six paired samples were selected covering the total range of quantification, from 2.14 log IU/ml to >7 log IU/ml. HBV was detected in 21 of 21 (100%) DBS from patients with a corresponding plasma viral load above 2.70 log IU/ml. The mean difference between plasma and DBS was 0.59 log IU/ml, and the correlation was strong (R2 = 0.92). In stability studies there was no significant change in DBS viral load after storage at room temperature for up to 12 weeks.

**Conclusions:**

This study suggests that DBS can be a feasible and reliable alternative to plasma for quantification of HBV in resource-limited settings. DBS can expand access to antiviral treatment for patients in low- and middle-income countries.

## Introduction

Globally, an estimated 240 million people are chronically infected with hepatitis B virus (HBV). In the absence of treatment, between 20 and 30% of patients with chronic HBV infection will progress to liver cirrhosis and/or hepatocellular carcinoma. The World Health Organization (WHO) estimates that 650,000 individuals die each year due to chronic hepatitis B (CHB) [1]. Although modern antiviral treatment of CHB effectively reduces the viral load and the risk of disease progression [2, 3], such treatment has been virtually unavailable in resource-limited settings up to now.

Measurement of hepatitis B viral load is essential in the management of CHB patients, both to determine whether antiviral treatment should be commenced, and to identify patients with treatment failure [4]. This test, however, requires a molecular laboratory with highly trained personnel and sophisticated technical instruments, as well as cold-chain facilities for storage of samples and reagents. Therefore, HBV DNA quantification is only performed at central laboratories, if at all, in low-income countries.

Dried blood spots (DBS) have been used for more than 40 years to screen for metabolic disorders in neonates [5]. The main advantage of DBS over plasma is that it can be stored at ambient temperature for weeks without degradation of nucleic acids [6], thus allowing transport of blood specimens from peripheral clinics to reference laboratories. Previous studies have shown that DBS can be used to reliably measure human immunodeficiency virus (HIV) RNA viral load [6–8] and hepatitis C virus (HCV) RNA viral load [9–11]. However, only a few smaller studies, and none in low-income countries, have assessed the performance of DBS for HBV DNA quantification and applied it in patient management [12–15].

In the present study, we used DBS collected at a routine CHB treatment clinic in Ethiopia to measure HBV DNA viral load and compared results with a gold standard plasma assay. Furthermore, we tested the stability of HBV DNA in DBS samples over time. If proven reliable DBS has the potential to improve access to HBV viral load—and thereby hepatitis B treatment—in resource-limited settings.

## Materials and Methods

### Patient samples

Participants were recruited from a CHB cohort at St. Paul’s Hospital Millennium Medical College, Addis Ababa, Ethiopia. All patients gave written informed consent to participate in the study. The study protocol conformed to the ethical guidelines of the 1975 Declaration of Helsinki, and was approved by The National Research Ethics Review Committee in Ethiopia and The Regional Committee for Medical and Health Research Ethics in Norway.

DBS were prepared in parallel with plasma from the same blood specimen. Plasma was collected and stored at -70°C. DBS samples were prepared by applying 80 μl of whole blood on each circle of a filter paper card (Whatman 903 sample collection cards, GE Healthcare Life Sciences, Norway) [16]. DBS were left to dry overnight and stored in sealed plastic bags with desiccants at room temperature for a median of 24 days (range 4–39) before shipment. Both plasma samples and DBS cards were shipped to the Norwegian Public Health Institute (Oslo, Norway) for analysis and stored at -80°C upon arrival.

### Extraction from DBS

DBS filter papers were taken out of -80°C and equilibrated at room temperature for minimum two hours. Three 6 mm diameter discs were punched from a single blood spot into a 1.5 ml tube. Two different extraction methods were used. The QIAgen DNA mini kit (Qiagen, Hilden, Germany) was used for the evaluation of DBS stability and variability together with an in-house real-time polymerase chain reaction (PCR). The Abbott RealTime HBV assay using the 0.2 ml protocol on sp2000 extractor (Abbott Molecular, Des Moines, IL, USA) and rt2000 real-time PCR instrument (Abbott Molecular, Des Moines, IL, USA) was used for comparison of hepatitis B viral load in DBS and plasma. In both cases, the lysis buffer for the respective extraction kits were used, i.e. DBS was dissolved in 180 μl ATL-lysis buffer with the Qiagen DNA mini-kit or 600 μl lysis buffer with the Abbott sp2000 kit.

Qiagen DNA mini-kit was used for viral DNA extraction from plasma (200 μl) and DBS according to the manufacturer’s recommendations with some modifications for the DBS protocol as follows: The DBS discs were incubated with ATL-lysis buffer at 85°C for 10 minutes. After addition of 20 μl of proteinase K, reaction tubes were vortexed for 30 seconds and subjected to incubation for one hour at 56°C in water bath. Then 200 μl of buffer AL was added to the reaction mix, vortexed and incubated at 70°C for 10 minutes. Thereafter, 200 μl of 100% ethanol was added and the mix was transferred to the QIAamp mini spin column, leaving filter paper debris in the tube. DNA was eluted in 100 μl of elution buffer. An initial comparison of the use of PBS pH 7.4 buffer and ATL-lysis buffer for extraction from DBS showed similar sensitivity (data not shown) so the ATL-lysis buffer from Qiagen was selected.

### Dilution factor between plasma and DBS

According to the manufacturer, each circle (diameter 13 mm, areal 133 mm^2^) of a Whatman 903 card holds 75–80 μl of whole blood [16]. A 6 mm disc (diameter 6mm, areal 28.3 mm^2^) is 21% of the 13 mm circle, which translates into 17 μl of whole blood. When using three 6 mm discs of dried blood, the input volume per analysis is 51 μl of whole blood. Since only plasma is used in viral load determination, we corrected for the hematocrit level by use of the median hematocrit (0.40) in the general population [17]. The following equation gives the input plasma per analysis: 51 μl * 0.60 = 30 μl. Hence, the dilution factor between DBS and plasma was 6.7 (200 μl: 30 μl) and 20 (600 μl: 30 μl) for extraction with the Qiagen and the Abbott systems, respectively.

### Stability testing of viral load on DBS

Three patients were selected for storage studies of DBS. All 3 had low to moderate viral load, near the recommended threshold for treatment eligibility. Several DBS cards were prepared from each patient and stored at room temperature over time. At time points 0, 2, 6 and 12 weeks, DNA was extracted from the DBS (3 parallels), as well as a sample control stored at -70°C. The extracts were stored at -70°C for 12 weeks, when all samples were analyzed with an in-house real-time PCR. The PCR was run twice on all samples.

### PCR

The in-house real-time PCR reactions were carried out in a total volume of 25 μl containing 3 mM of Mg, 0.2 mM of dNTPs, 0.2 μM each of primers, 250 nM of TaqMan probe, 2 units Pt taq (Invitrogen, Thermo Fisher Scientific, MA, USA) and 10 μl DNA extract. The primer and probe sequences were as follows; 5’ GGCACTAGTAAACTGAGCCA 3’ (forward), 5’ GTATGTTGCCCGTTTGTCCTC3’ (reverse) and 5’ CGGRCTGAGGCCCACTCCCATAGG3’ (TaqMan-probe) labeled with FAM/BHQ1. The qPCR cycling conditions were at 96°C for 2 minutes, 45 cycles at 94°C for 10 seconds and 68°C for 30 seconds with acquired at 82°C followed by a melting curve analysis using Rotor-Gene 6000 (Qiagen, Hilden, Germany). The limit of detection (LOD) for the in-house qPCR was estimated to be at least 250 IU/ml based on serial dilutions of a well characterized and quantified patient sample (genotype C).

Paired plasma and DBS from 26 patients were quantified using the DNA protocol (0.2 ml) on Abbott m2000sp and quantified with the Abbott RealTime HBV assay on the Abbott m2000sp with a LOD of 15 IU/ml.

### Statistical analysis

DBS and plasma HBV DNA levels were compared by linear regression analysis on log10 transformed data, and the mean difference was analyzed with paired samples t-test. In order to describe agreement between plasma and DBS results, the analysis described by Bland and Altman was used [18]. Data were analyzed using SPSS version 21.0 for Windows (SPSS Inc., Chicago, IL, USA), except 95% confidence intervals for proportions which were calculated with OpenEpi version 3 (OpenEpi, Atlanta, GA, USA).

## Results

### Quantification of HBV DNA using DBS

HBV DNA was quantified in paired plasma and DBS on a selection of 26 samples from hepatitis B patients with viral load values covering the quantification range from 2.14 log to >7 log IU/ml using plasma as reference. HBV DNA was detected in 23 of 26 (88%) DBS samples (Table 1). Three of the positive samples were below the limit of detection when analyzed on DBS. Among 5 samples with a low viral load (<2.70 log IU/ml), there were 3 false negative DBS results with a corresponding plasma viral load of 2.14, 2.40 and 2.68 log IU/ml, respectively. Two DBS samples within this same range were positive with a corresponding plasma viral load of 2.58 and 2.66 log IU/ml.

**Table 1:** Plasma viral load and corresponding HBV DNA detection rates in DBS.

Plasma viral load	Plasma (n = 26)	DBS (n = 26)	DBS detection rate (95% CI)
<2.7 log IU/ml	5	2	40% (12–77)
> 2.7 log IU/ml	21	21	100% (85–100)

DBS, dried blood spots; CI, confidence interval.

### Correlation between DBS and plasma viral load results

**Fig 1:**
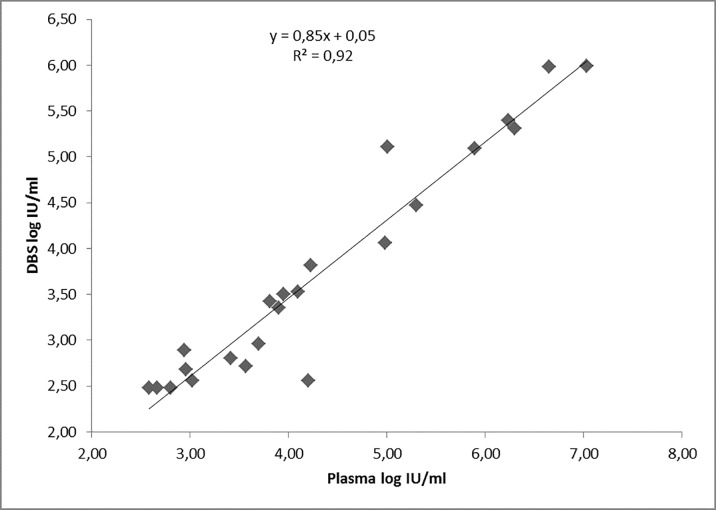
Regression analysis of HBV DNA levels in 23 paired plasma and Dried Blood Spot (DBS) specimens from Ethiopian hepatitis B patients

Linear regression analysis was performed to determine the relationship between the HBV viral load obtained from plasma and DBS. A strong correlation (R^2^ = 0.92) in viral load values was obtained for the 23 paired samples analyzed (Fig 1).

**Fig 2:**
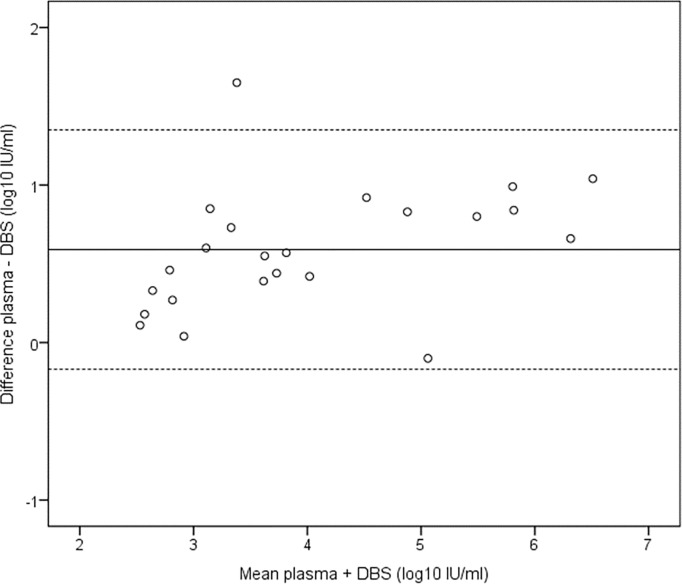
Bland and Altman analysis of agreement between HBV DNA levels in 23 paired plasma and Dried Blood Spot (DBS) specimens. The horizontal lines represent the mean difference (continuous line) and +/- 1.96 standard deviations (dotted lines).

The viral load was significantly higher in plasma than DBS; mean difference was 0.59 log IU/ml (95% confidence interval [CI], 0.42–0.75, p <0.001). The Bland-Altman plot illustrates the agreement between plasma and DBS viral load results (Fig 2).

### Stability and variability of HBV viral load in DBS

**Fig 3:**
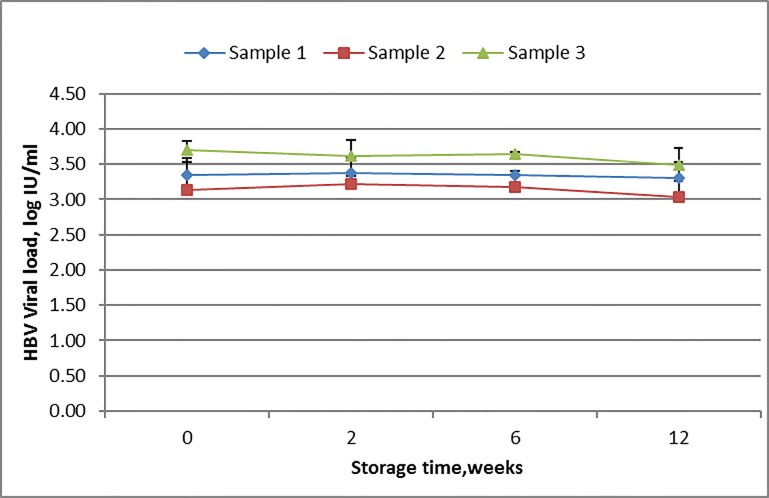
HBV DNA levels in Dried Blood Spots (DBS) after 2, 6 and 12 weeks of storage at room temperature.

The stability of hepatitis B viral load in DBS during storage at room temperature over time was evaluated using DBS from 3 patients with a low to moderate viral load. Three parallels of each sample were analyzed at each time point. The viral load was stable at room temperature for up to 12 weeks with a coefficient of variation of less than 5% (Fig 3). When all extracts were repeated on PCR the coefficient of variation was less than 10%, indicating low intra-assay variability.

## Discussion

In this study, we examined the viral load in paired DBS and plasma using a commercial test for quantification of HBV. There was good concordance between HBV viral load in plasma and DBS over a dynamic range of viral loads when adjusted for the dilution factor between plasma and whole blood on DBS. However, the mean viral load was approximately 0.59 log IU/ml lower in DBS compared to plasma.

The LOD for DBS in combination with the Abbott RealTime HBV assay appears to be around 2.7 log IU/ml (= 500 IU/ml). This is, as expected, higher than when analyzing plasma due to a much lower sample volume in DBS. This has also been reported in other studies and depends both on the extraction process and input volume [13, 15, 19]. The sensitivity of DBS may be improved by increasing the DBS sample volume in the extraction protocol, for example by using several blood spots, but it is not likely to reach the same level as when using plasma. Consequently, samples with a viral load below 2.7 log IU/ml may be false negative using a DBS protocol, and DBS should not be used in settings where even a low viral load may be clinically relevant, such as blood transfusion screening, or to detect occult infections.

For the assessment of treatment eligibility in chronic hepatitis B patients, on the other hand, DBS might indeed be useful. International treatment guidelines recommend to start antiviral treatment in individuals with signs of continued necro-inflammation in the liver if HBV DNA viral load exceeds 3.3 log IU/ml (= 2000 IU/ml) [20].These patients are at risk of progression to cirrhosis and hepatocellular carcinoma in the absence of antiviral treatment. Inactive carriers, characterized by HBV DNA levels <3.3 log IU/ml, however, have an excellent prognosis in the absence of treatment [21, 22]. In this context the use of DBS has a satisfying sensitivity to detect those in need of treatment.

DBS might also be a suitable tool for monitoring antiviral treatment effect. Patients who receive standard first line therapy with tenofovir or entecavir will effectively suppress the viral load to undetectable levels [23]. Treatment failure is detected either by absence of >1 log IU/ml initial viral load decline or by a >1 log IU/ml rise in viral load after an initial antiviral treatment effect [1]. Our study suggests that DBS will be sufficiently sensitive to be used in clinical patient management, since the variable of interest is the change in viral load rather than the absolute number. Thus, DBS probably has an adequate sensitivity to detect viral breakthrough of clinical relevance, and the frequency of sampling during monitoring might be a more important factor for early detection of viral breakthrough than the sensitivity of the test. Of note, the same test should be applied in monitoring viral load during treatment due to variability between quantitative tests.

We chose to test the stability of samples with low to moderate viral load stored at room temperature in order to simulate realistic conditions in resource-limited settings. The viral load showed no significant decline when stored at room temperature for up to 12 weeks. To our knowledge, this is the only study that has evaluated the stability of HBV DNA viral load on DBS using whole blood and over such a long time. The good stability of HBV DNA over time makes DBS applicable for HBV management even in remote settings, where samples can be easily collected, stored and transported to reference laboratories for analysis without need for special handling or cold-chain facilities.

Our study has certain limitations. First, the sample size was restricted and the exact LOD of the DBS protocol cannot be accurately ascertained based on our results alone. Second, the limited number of DBS per patient also limited the assessment of intra- and inter-assay variability using the DBS protocol. Third, the stability study was carried out using samples with low to intermediate viral load around the threshold for treatment indication. Since higher concentrations of virus are generally more stable than lower concentrations, the data should be interpreted with care if extrapolated to other levels. The main strength of our study was that we tested the use of DBS in a real-life setting in a low-income country. Therefore, we believe our findings are more relevant than laboratory based studies carried out in high-income countries.

In summary, we found that DBS for HBV viral load testing is a good alternative to plasma as it is stable during storage at room temperature and therefore allows easy handling, storage and transport of specimens in rural areas. The sensitivity is lower in DBS compared to plasma, but the viral load correlates well with a gold standard plasma assay. For the management of CHB, DBS appear to be sufficiently sensitive to detect patients in need of treatment, as well as those who experience treatment failure. The ideal solution for HBV DNA monitoring would be a robust, reliable and inexpensive point-of-care assay; however, this is still not commercially available. In the meantime, DBS can improve access to viral load measurements and thereby antiviral treatment in resource-limited settings, and is currently the only viable option for HBV DNA testing in many parts of the world.
